# Image-Based Lunar Hazard Detection in Low Illumination Simulated Conditions via Vision Transformers

**DOI:** 10.3390/s23187844

**Published:** 2023-09-13

**Authors:** Luca Ghilardi, Roberto Furfaro

**Affiliations:** University of Arizona, Tucson, AZ 85721, USA; lucaghilardi@arizona.edu

**Keywords:** image segmentation, vision transformer, deep neural network, supervised learning, hazard detection, lunar south pole, lunar hazard detection, vision-based hazard detection

## Abstract

Hazard detection is fundamental for a safe lunar landing. State-of-the-art autonomous lunar hazard detection relies on 2D image-based and 3D Lidar systems. The lunar south pole is challenging for vision-based methods. The low sun inclination and the terrain rich in topographic features create large areas in shadow, hiding the terrain features. The proposed method utilizes a vision transformer (ViT) model, which is a deep learning architecture based on the transformer blocks used in natural language processing, to solve this problem. Our goal is to train the ViT model to extract terrain features information from low-light RGB images. The results show good performances, especially at high altitudes, beating the UNet, one of the most popular convolutional neural networks, in every scenario.

## 1. Introduction

As interest in returning to the Moon grows, the challenge of safely landing on the lunar surface becomes increasingly essential. NASA’s ALHAT (Autonomous Precision Landing and Hazard Detection and Avoidance Technology) [1,2,3] program is developing new technologies for lunar landing guidance, navigation, and control to support both manned and unmanned missions. The Apollo 11 mission highlighted the difficulty of the landing task and the potential risks, as testified by the fact that Neil Armstrong had to navigate the spacecraft to avoid a dangerous crater manually. To address these challenges, NASA’s Vision for Space Exploration program emphasizes the need for increased autonomy during landing maneuvers, particularly in hazard detection and avoidance [4]. These functions are already being performed autonomously, as demonstrated by the Chinese Chang’e 3 mission.

The Chang’e 3 was the first robotic spacecraft that adopted autonomous hazard detection and avoidance technology. It represents the state of the art regarding autonomous lunar landings as it has achieved the highest landing precision. The first Chang’e 3 hazard detection design included only the visual-based hazard detector, but it failed to pass validation tests. The final state of the system had a combination of two-dimensional (2-D) optical gray-image-based coarse hazard avoidance and three-dimensional (3-D) laser-elevation-image-based [5,6]. The vision-based hazard detection was used to perform a coarse hazard detection from below 2 km to around 100 m. Coarse avoidance aimed to exclude hazardous large-scale obstacles (craters and boulders larger than 1 m) and to provide potential safe landing regions for the following precise avoidance. At around 100 m, the lander entered a hovering phase, during which it scanned the surrounding area with a LiDAR (light detection and ranging). Next, the onboard guidance-navigation and control (GNC) system detected craters larger than 20 cm and slopes steeper than 8 deg (10 m baseline) and determined the nearest safe landing site. The following Chang’e 4 and 5 landers used a similar design to Chang’e 3 [7,8], confirming the robustness of the design.

To the authors’ knowledge, machine learning-based hazard detection has yet to be exploited for real planetary landing missions. Hazard detection has typically been carried out using more traditional methods. Among these methods, there are the Canny [9,10] and Sobel [11] algorithms, which detect the edges in an image, and the various corner detection algorithms [12,13], which can provide good repeatability along with localization [14]. However, these methods may not meet the accuracy and computational efficiency requirements for real-time use on a spacecraft hazard detection system. Many machine learning models have been studied to address these challenges as they can provide high accuracy with relatively low computational time. For image-based hazard detection in particular, some machine learning classifiers, such as perceptrons [15], have been explored. Still, these approaches generally require the manual input of relevant features of the surface. In contrast, the proposed approach aims to automate feature extraction by using deep learning techniques such as semantic segmentation to classify safe and unsafe areas of the lunar surface. In the past, the authors and other research groups explored the capabilities of the UNet [16] architecture applied to the lunar landing hazard detection problem [17,18,19]. Based on a deep convolutional encoder/decoder (DCE) structure, this architecture is known for its versatility and high accuracy in various applications [20,21,22].

In recent years, transformer architecture has been proven to be a breakthrough in the natural language processing (NLP) field [23]. Based on that architecture, the vision transformer (ViT) encoder is born [24], achieving state-of-the-art results on various computer vision tasks, such as image classification and object detection. At the same time, DCEs are typically used for low-level vision tasks, such as image processing. The key advantages of ViT architecture are its ability to handle high-resolution images, variable-size inputs, and better generalization, maintaining a global receptive field throughout the encoder. The authors tested this architecture in a monocular depth-estimation problem with good results [25].

This paper presents an image-based hazard detection algorithm exploiting vision transformer for spacecraft 6-DOF models, as well as considering different lighting conditions. We modified the architecture explored in [26] for a classification task. First, the encoder is designed according to the architecture proposed and pre-trained on the ImageNet dataset. Subsequently, we fine-tune the encoder on a custom lunar landing hazard detection dataset. The dataset (https://data.cyverse.org/dav-anon/iplant/home/ghila/Dataset_Hazard.tar.gz, accessed on 20 July 2023) used to train and test the network is created using the 3D model software, Blender 3.2 (Blender is open-source software for modeling and physically based rendering), and it considers a 6-DOF model of the spacecraft, which poses additional difficulties in autonomous image-based hazard detection due to different lighting conditions and the observed shapes of the craters.

The manuscript is organized as follows. First, the architecture of ViT is described together with the simulation environment and the dataset creation. Afterward, some indices are introduced to test the model’s performances. Finally, concluding remarks are given.

## 2. Method

**Figure 1:**
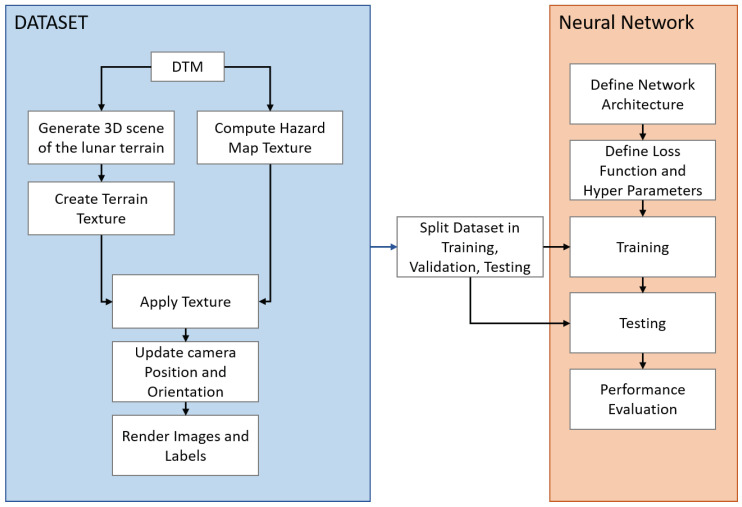
Workflow diagram for the dataset creation and network training and testing. The optical images are simulated starting from a digital terrain model (DTM) of the south pole.

The architecture and dataset creation are described in this section. In Figure 1, the workflow diagram is presented.

### 2.1. Architecture

**Figure 2:**
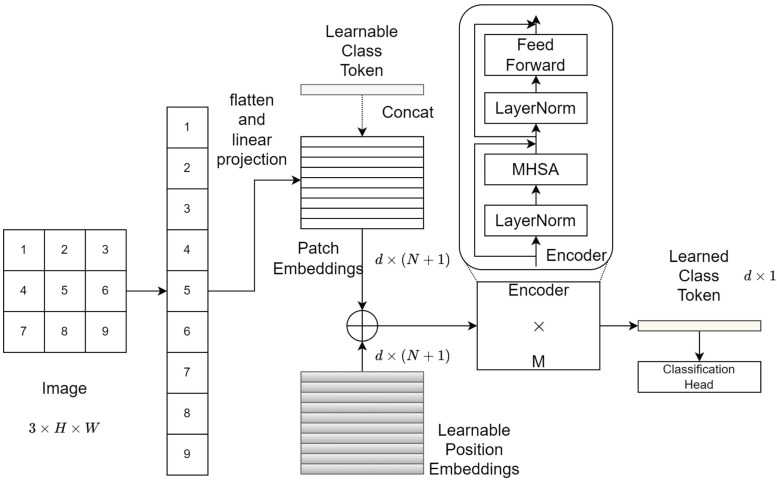
The network architecture of ViT [27].

The goal of this section is to introduce ViT architecture. As previously mentioned, one of the transformer encoder’s main advantages is maintaining a global receptive field with high feature resolution throughout the encoder without loss of information. On the other hand, in a typical convolutional neural network, increasing the receptive field in the deepest layers of the encoder comes at the cost of the resolution of the image and thus a loss of detail. The vision transformer layers were first introduced by Dosovitskiy et al. [24] as a method based on the original transformer of Vaswani et al. [23] for image classification, Figure 2. Here, we adopted the architecture created by Belkar et al. (https://github.com/antocad/FocusOnDepth, accessed on 20 July 2023), which is more customizable and easier to understand.

Before being input into the transformer encoder, the image $X\in R^{h\times w\times c}$ is subdivided into non-overlapping patches $X_{p}\in R^{N\times (p^{2}\cdot c)}$ called tokens. *h* and *w* are the spatial image resolution, *c* is the number of channels, (p,p) represents the patch size, and $N=hw/p^{2}$. Since the transformer uses constant latent vector size and is position invariant, the tokens are linearly embedded into a feature space *D* to identify the position and class of each token. This allows the model to weigh each patch’s attention with respect to its position.

(1) $$z_{0}=\left[x_{class};x_{p}^{1}E;x_{p}^{2}E;...;x_{p}^{N}E\right]+E_{pos}\;\mathrm{where},\;E\in R^{(p^{2}\cdot c)\times D},\;E_{pos}\in R^{(N+1)\times D}$$

Once embedded, the patches are passed into the transformer encoder, which returns a value for each class token. As shown in Figure 2, the embedded patches become normalized and are fed into the multi-head attention block. Before explaining how this block works, we need to understand the self-attention mechanism [28].

The vectors derived from different inputs are packed in three matrices: queries Q, keys K, and values V. Each query is compared with the keys to obtain scores/weights for the values. Each score/weight is, in short, the relevance between the query and each key. One can reweight the values with the scores/weights and complete a summation of the reweighted values. The attention function can be broken down into the following steps:

1 Compute scores $S=Q\cdot K^{T}$, which measures the degree of attention of the surrounding image patches.
2 Normalize the scores for the stability of the gradient $S_{n}=S/\sqrt{D}$
3 Translate the scores into a probability with the softmax function $P=softmax(S_{n})$
4 Compute weighted value matrix Z=P·V

The function can be expressed as follows:

(2) $$\mathrm{Attention}\left(Q,K,V\right)=\mathrm{Softmax}\left(\frac{Q\cdot K^{T}}{\sqrt{D}}\right)\cdot V$$

Multihead self-attention (MHSA) is an extension of self-attention to boost its performance, where we run *k* self-attention operations in parallel.

(3) $$\begin{array}{cc} \mathrm{MultiHead}(Q^{\prime },K^{\prime },V^{\prime }) & =\mathrm{Concat}\left(head_{1},head_{2},...,head_{h}\right)W_{0}\;W_{0}\in R^{D\times D} \end{array}$$

(4) $$\begin{array}{cc} \mathrm{where},\;head_{i} & =\mathrm{Attention}(Q_{i},K_{i},V_{i}) \end{array}$$

$Q^{\prime },K^{\prime }$, and $V^{\prime }$ are the concatenation of $Q_{i}^{m},K_{i}^{m},V_{i}^{m}$, where *m* is the number of parallel MHSA layers. At last, $W_{0}$ is the projection weight. Following the design of the original transformer [23], after the self-attention layer, a feed-forward network (FFN) is applied to introduce non-linearities with activation functions. It is essential to model complex relationships between the elements.

The encoder adopted is the ViT-Base [24] that has been pre-trained on ImageNet [29]. Most importantly, for MDE applications, the transformer encoder does not use down-sampling operations, which preserve all the image details. Into the encoder, multiple transformers layers are applied on the cascade. Once the encoder has processed the tokens, they must be reassembled to complete the dense prediction.

**Figure 3:**
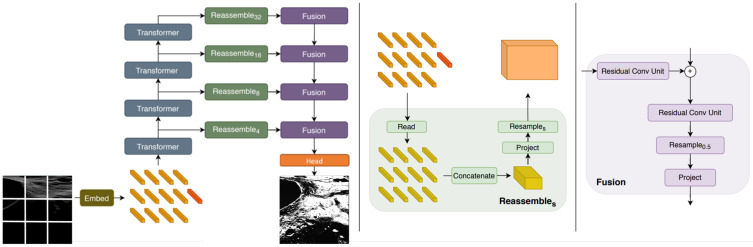
Vision transformer network architecture. This picture is a modified version of the architecture overview presented in [26].

As shown in Figure 3, the encoder has representation extractions. These representations are combined in the reassemble block, which transforms such representations into a higher space that can be used in the fusion block. Each reassemble block is composed of 3 sub-blocks:

- Read block: this reads the input to be mapped into a representation size, by concatenating the readout token. The readout token is generally responsible for aggregating information from other tokens [30]. However, in the case of vision transformers, their performance is limited.
  (5) $$R^{(N+1)\times D}\to R^{(N)\times D}$$
- Concatenate block: in this block, the representations are combined. The step consists of concatenating each representation following the order of the patches. This yields an image-like representation of this feature map.
  (6) $$R^{(N)\times D}\to R^{\frac{h}{p}\times \frac{w}{p}\times c}$$
- Resample block: This block consists of applying a 1 × 1 convolution to project the input image-like representation into a space $\hat{D}$ of dimension 256. This convolutional block is followed by another 3 × 3 convolution to implement spatial downsampling and upsampling operations.
  (7) $$R^{\frac{h}{p}\times \frac{w}{p}\times c}\to R^{\frac{h}{s}\times \frac{w}{s}\times \hat{D}}$$
  where *s* is the chosen scale size of the representation.

The fusion block takes the representations from the reassemble and the previous fusion block to sum them. When they are summed, we apply two successive convolutional units and upsample the predicted representations. A more detailed description of the decoder can be found in [26].

### 2.2. Dataset

The dataset comprises 125,000 images of the lunar south pole, specifically in an area of 90×90 km around the southern point. The images have been created using open-source 3D modeling software called Blender. Within Blender, we load a digital terrain model (DTM) of the area of interest provided by NASA (https://pgda.gsfc.nasa.gov/products/81, 10 October 2022) [31].

**Figure 4:**
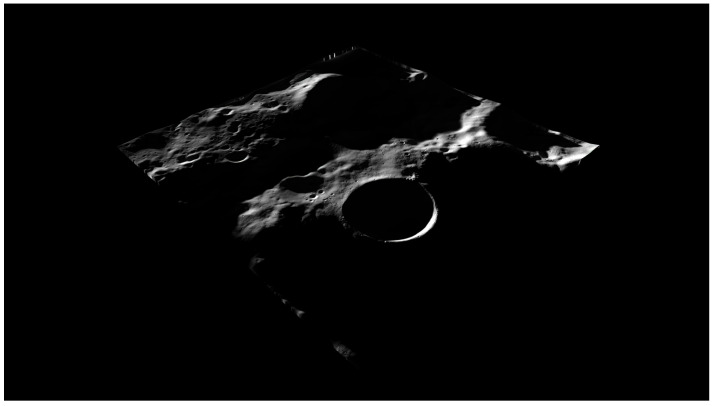
Image of the surface 3D model.

The model has been reduced in size to accommodate the memory specifics of our machine. To simulate the terrain, we create a plane of the same size as the DTM. Then, thanks to Blender’s function “displacement map”, the terrain features are made. Once we assign a texture of the color of the regolith as a material and a light source, Blender uses a bidirectional scattering distribution function (BSDF) model to compute how each light ray interacts with the terrain and the surrounding objects, Figure 4. This allows Blender to render photo-realistic images and recreate some photometric functions. The scene’s illumination is provided by a “sun lamp” with an inclination of 1.5 deg, with the terrain rotated randomly around the azimuth axis [32]. It is worth noticing that our model does not consider the Moon curvature, i.e., the terrain elevation of the DTM is with respect to a flat plane. Once the scene has been set up, we create a list of positions and inclinations for the camera. Specifically, the position components are taken in a plane grid of 10×10 km and from a maximum altitude of 10 km to a minimum of 500 m above the terrain. The camera’s inclination is set up randomly up to a maximum value that depends on the respective position altitude. This has been done to prevent the camera’s frame from seeing the border of the DTM. Up to 4 km, the inclination to the nadir is up to a maximum of 45 deg. From 4 to 9 km, the maximum value is 30 deg, and above 9 km, the maximum value is 20 deg. At each position, an image is rendered from the camera frame. No noise effects [33] have been considered during the dataset created for this work.

**Figure 5:**
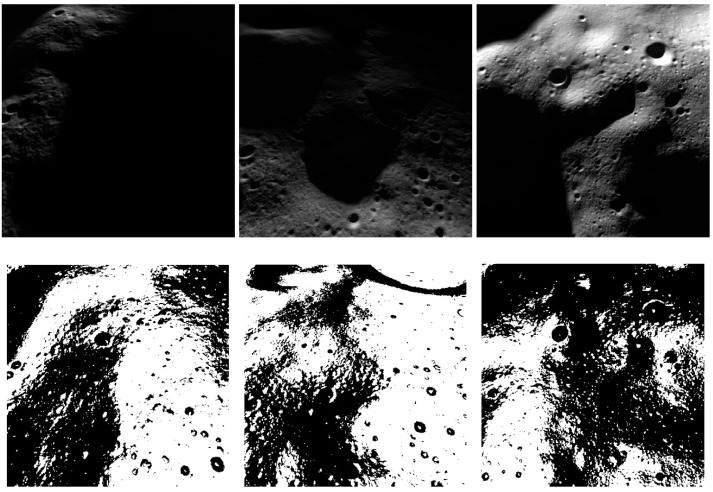
Samples of the dataset. In the first row, the input images are shown; in the second, the labels.

Since we are doing supervised training, we also need the corresponding ground truth associated with each image. To facilitate the process, Blender allows one to superimpose an image texture to the terrain. Therefore, we render the respective ground truth in addition to the image, modifying the illumination conditions to avoid unwanted shadows. The ground truth texture image is created by combining the requirements of maximum terrain slope and roughness for a safe landing site. The terrain slope is computed pixel-wise with the local gradient of the DTM, while the local roughness has been computed with a standard deviation filter. In the ground truth, a pixel is considered safe if the slope of that pixel is less than 8 deg and the roughness is less than 10%. Figure 5 shows a few examples of rendered images with the respective labels. For the labels, the pixels in “black” are the hazardous pixels, and the ones in “white” are safe.

The images are rendered with a resolution of 384×384 in RGB with a camera with a focal length of 50 mm. The dataset has been augmented using random rotations between −10 and +10 degrees, random image crops with a minimum resolution of 256×256, and by flipping the images.

Then, 60% of the dataset is randomly selected for the training, 20% is selected for the validation, and the last 20% is used for testing.

It is worth mentioning that the camera model in Blender works differently from a real CMOS sensor. This gap can be mitigated, as shown in [34]. However, this method requires real measurements or very accurate lab data to create a realistic photometric model [35], which the authors do not have access to at this time. This reason, combined with the lack of noise in the images, does not allow the authors to make any claim on how the dataset compares with real data.

## 3. Parameters

The training has been performed using the ADAM optimizer with a learning rate set to be 1 × 10${}^{-5}$ applied to the cross-entropy loss function. We used a batch size of 5 and the ViT-base backbone. The encoder weights are initialized on the ImageNet trained checkpoint. Then, the decoder is trained from scratch. The model has been trained for 100 Epochs. The parameters selected are the ones that gave the best validation loss and overall loss behavior on multiple training runs with a reduced number of epochs.

## 4. Metrics

The following methods and terminology are employed to evaluate the test data:

- TP = true positive, data points predicted as hazardous that are hazardous.
- TN = true negative, data points predicted as safe that are safe.
- FP = false positive, data points predicted as hazardous that are safe.
- FN = false negative, data points predicted as safe that are hazardous.

The metrics adopted for the proposed semantic segmentation experiments are precision vs. recall and the intersection over union (IoU), which are described next.

### 4.1. Precision vs. Recall

The precision vs. recall is more suitable than the global accuracy metrics for semantic segmentation because the latter will yield misleading results if the data set is unbalanced. In our case, the hazardous terrain must be correctly classified because any hazard can lead to mission failure. Therefore, we need a metric that takes into account the FN.

The recall refers to the percentage of total relevant results correctly classified by the algorithm, and it is computed as:

(8) $$Recall=\frac{TP}{TP+FN}$$

Conversely, precision refers to the percentage of the relevant results:

(9) $$Precision=\frac{TP}{TP+FP}$$

As we discussed earlier, the FN are extremely important because they can lead to mission failure. Consequently, high recall is desirable. Nevertheless, one should avoid low precisions to avoid situations where the algorithm does not clearly identify any safe landing site.

### 4.2. Intersection over Union

The intersection over union score, also named the Jaccard index, is often used to compare multi-class semantic segmentation methods. It is defined as follows:

(10) $$IoU=\frac{TP}{TP+FP+FN}$$

Compared to precision and recall, the IoU is a stricter metric because it considers TP, FP, and FN all together. The latter means that the IoU score results in lower scores since every misclassified pixel considerably impacts the overall score. We compute the IoU score for each class separately and compute the mean of all class scores afterward.

## 5. Results

After training, the vision transformer model is evaluated on the test set. The test set is 20% of randomly selected pictures in the training set, for a total of 25,000 images spanning from a minimum height of 500 m to a maximum of 10 km. The model performance is also been compared with a selected UNet’s architecture defined in Table 1. Generally, we observed good performance from the UNet model even with small datasets [17]. The UNet has been trained on the same dataset of the ViT with the same data augmentation. We employed the same cross-entropy loss function using the ADAM optimizer with a learning rate of 1 × 10${}^{-5}$, a batch size of 20, and 100 epochs. However, we saved the model weights only when the validation loss decreased to the previous epoch. The validation minimum was at the 35th epoch, meaning the model was overfitting in subsequent iterations.

Both models are trained with the GPU CUDA system to accelerate the processes. The code has been written in Python 3.9, exploiting the machine learning tools provided by the Pytorch package.

**Figure 6:**
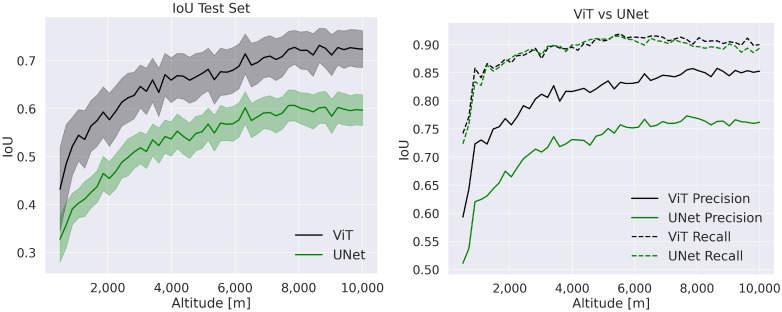
IoU plot on the test set. The transparent area in the IoU plot represents the standard deviation.

Figure 6 shows the results of both models on the test set. The values in these plots are the average values of pictures at a specific altitude. Apparently, both models perform better at higher altitudes. This may happen because the images taken at higher altitudes have more chance to show illuminated areas, thus making relevant terrain features visible. The standard deviation is the transparent area on the right plot around each line. Also, in this case, we notice a smaller standard deviation at higher altitudes. The ViT model outperforms the UNet for both IoU and precision metrics. However, the recall values for both models are very similar, thus stressing the importance of employing both precision and recall as evaluation metrics. Here, we conclude that the UNet model classification is biased toward hazards.

**Figure 7:**
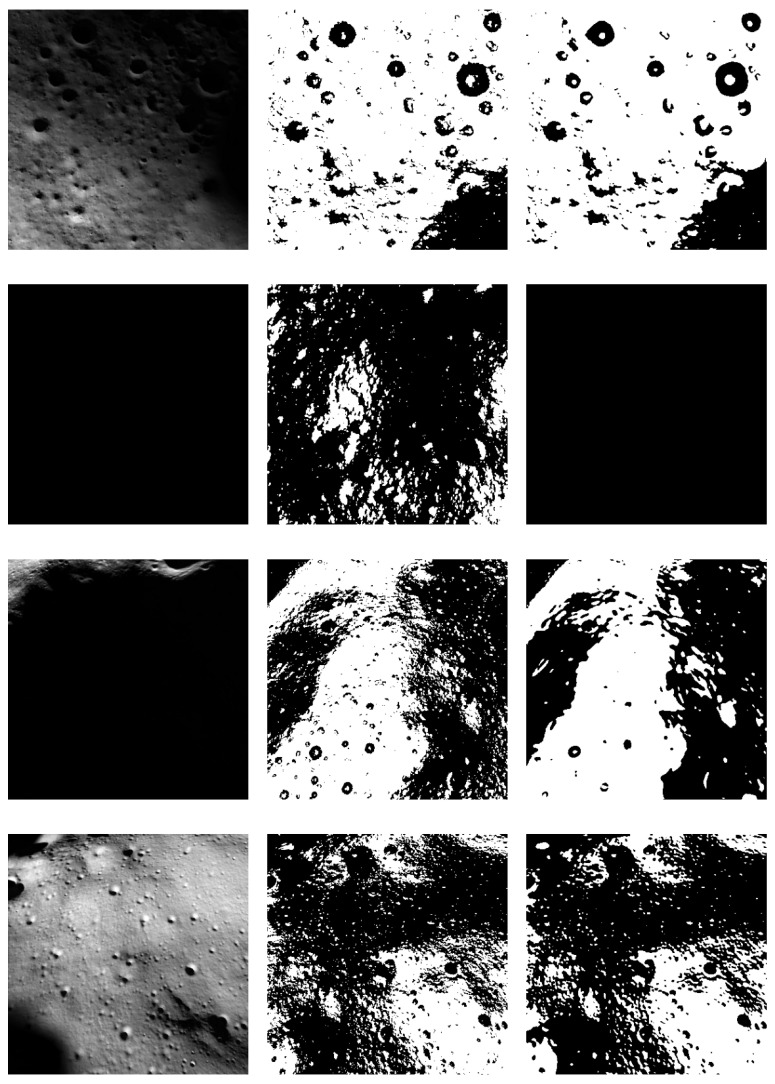
In the sample test dataset, the input images are shown in the first row; in the second, the ground truth; and in the third row, the Vision Transformer model’s predictions. The “white” pixels are labeled as safe, while the “black” ones are labeled as hazardous.

The relationship between model performance and the altitude of the image can be additionally established by qualitatively analyzing the test results. In some images, the camera does not capture any light (dark crater spots), making the model incorrectly classify the whole image as hazardous. In Figure 7, some examples of the test set classified by the ViT model are shown.

**Figure 8:**
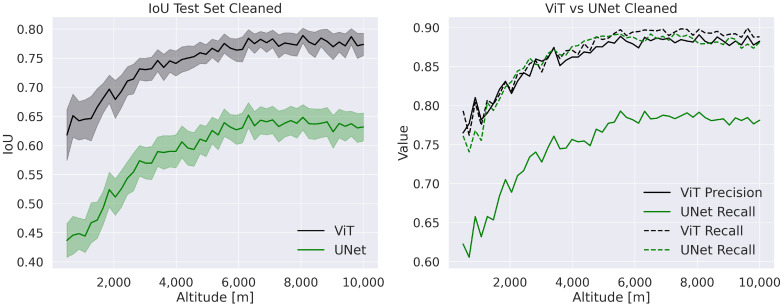
IoU plot on the test set “cleaned”. The transparent area in the IoU plot represents the standard deviation.

Including these completely black images makes the test set closer to a real-life scenario. However, obtaining a reliable output from input without information is virtually impossible. Consequently, in Figure 8 we show the ViT performance on a “cleaned” test set to evaluate the model performance when black images are absent. More specifically, 4891 images out of the 25,000 of the test set have been discarded. Both models’ performance mainly benefits by filtering out the images with no information at lower altitudes. We also notice that the standard deviation on ViT’s IoU value is smaller, thus indicating higher robustness.

**Figure 9:**
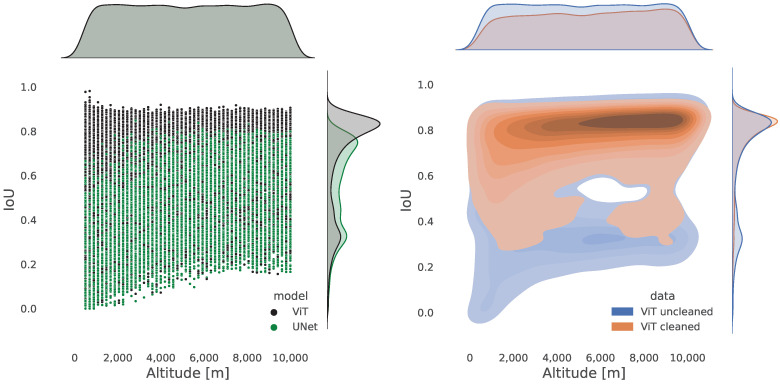
On the left, the IoU distribution for the ViT and UNet models. On the right, the IoU distribution for the ViT model on the “cleaned” and “uncleaned” datasets.

Figure 9 shows the distribution of the test set images with respect to the altitude and the distribution of the IoU score. On the left plot, one can see the distribution of the IoU score of the ViT as compared to the UNet on the test set. The test images are normally distributed in the “uncleaned” dataset. Conversely, on the right plot, we notice a reduction in the number of images at lower altitudes in the “cleaned” dataset. What concerns the distribution of the IoU score is a reduction in the number of lower scores, which are associated with images with no information. On the right plot, the distribution peak for the IoU value is around 80% for an altitude that spans between around 5000 and 9000 m.

Both models are not optimized for real-world deployment. However, it is interesting to see how the two models compare on computational speed. The mean time for one iteration (one image) in the ViT model is 0.346 s, with a maximum time of 0.500 s and a minimum of 0.318 s. The UNet shows a faster computational time with a mean time of 0.012 s, a maximum time of 0.054, and a minimum of 0.006. Both models have been trained and tested on a machine with the following specifics: dual CPU Xeno E5-2680 v4, 256 Gb of RAM, and GPU Nvidia RTX3090.

## 6. Conclusions

In this work, we designed and evaluated the performance of a vision transformer architecture for hazard detection during the Spacecraft’s descent toward the lunar surface in the challenging case of low illumination conditions. Vision transformers are a new architecture for image encoders, where the image is split into fixed-size patches. Each of them is then linearly embedded, position embeddings are added, and the resulting sequence of vectors is fed to a standard transformer encoder. The ViT model represents an input RGB image as a series of image patches and directly predicts class labels for the image. The dataset comprises images of the lunar south pole surface with the corresponding segmented labels “hazardous” or “safe” depending on the pixel’s local slope and roughness values. The images have been created using the lunar south pole DTM in the modeling/rendering software Blender.

The ViT model performs well in recognizing hazards at altitudes ranging from 5000 to 9000 m, even in low-light conditions. The authors also found that the ViT model outperforms a classic UNet model. Compared to a classical convolutional network, the ViT encoder does not downsample the input image. Therefore, there is no loss of information to learn the terrain features maintaining the global receptive field throughout the training. ViT performance is at its best between 5000 and 9000 m, and the metrics values degrade as the camera gets closer to the surface. It is important to note that images without information poison the metrics since the camera sensor did not gather enough light. Nevertheless, the inclusion of these images is a more realistic scenario.

It is worth noticing that no noise has been added to the images. The ViT network has more learning parameters than the UNet, specifically 105 million vs. 17 million of the UNet, which influences both training and deployment computational speed.

Low-light computer vision with a passive sensor is a challenging problem. This model cannot substitute an active sensor like LiDAR, independent of the illumination conditions. Nevertheless, the model is powerful at very high altitudes and is easy to implement as a backup or aid system to the more complex sensors.

The authors plan for the future is to refine the dataset by adding environmental and camera noises and adopt a smaller and less computationally intensive ViT model on an Nvidia Jetson Nano for a test with a real camera and a lunar mocap.

## Figures and Tables

**Table 1:** UNet architecture.

Blocks	Channels
Double Conv.	3 → 64
Down1	64 → 128
Down2	128 → 256
Down3	256 → 512
Down4	512 → 1024
Up1	1024 → 512
Up2	512 → 256
Up3	256 → 128
Up4	128 → 64
Classification Head	64 → 2
**Blocks**	**Layers**
Double Conv.	Conv2D
	BatchNorm2D
	ReLU
	Conv2D
	BatchNorm2D
	ReLU
Down	MaxPool2D
	Double Conv.
Up	Bilinear Up-Sampling
	Double Conv.
Classification Head	Conv2D

## Data Availability

The dataset created and adopted for this work is available at: https://data.cyverse.org/davanon/iplant/home/ghila/Dataset_Hazard.tar.gz.

## Abbreviations

The following abbreviations are used in this manuscript:

Lidar	Light detection and ranging
ALHAT	Autonomous precision landing and hazard detection and avoidance technology
GNC	Guidance-navigation and control
DCE	Deep convolutional encode
NLP	Natural language processing
ViT	Vision transformer
DOF	Degrees of freedom
MHSA	Multihead self-attention
FFN	Feed-forward network
BSDF	Bidirectional scattering distribution function
DTM	Digital terrain model
TP	True positive
TN	True negative
FP	False positive
FN	False negative
IoU	Intersection over union
